# Novel Bispecific Engagers Targeting the CεmX Domain of mIgE‐Expressing Cells

**DOI:** 10.1111/all.70236

**Published:** 2026-02-02

**Authors:** Constantin Möller, Julia Hambach, Alessa Zoe Schaffrath, Thomas Eden, Sophia Cichutek, Anna Josephine Gebhardt, Natalie Tode, Ronald Maitschke‐Rajasekharan, Philipp Lewe, Sharon Qi, Susanne Witt, Marie Eggers, Nicola M. Tomas, Friedrich Haag, Friedrich Koch‐Nolte, Boris Fehse, Kristoffer Riecken

**Affiliations:** ^1^ Research Department Cell and Gene Therapy, Department of Stem Cell Transplantation University Medical Center Hamburg‐Eppendorf (UKE) Hamburg Germany; ^2^ Institute of Immunology University Medical Center Hamburg‐Eppendorf (UKE) Hamburg Germany; ^3^ Department of Oncology, Haematology and Bone Marrow Transplantation with Section Pneumology University Medical Center Hamburg‐Eppendorf Hamburg Germany; ^4^ University Medical Center Hamburg Eppendorf (UKE) Hamburg Germany; ^5^ Center for Structural Systems Biology Hamburg Germany; ^6^ European Molecular Biology Laboratory Hamburg Hamburg Germany; ^7^ III. Department of Medicine University Medical Center Hamburg‐Eppendorf (UKE) Hamburg Germany; ^8^ Hamburg Center for Kidney Health (HCKH) University Medical Center Hamburg‐Eppendorf Hamburg Germany; ^9^ Hamburg Center for Translational Immunology (HCTI), UKE Hamburg Germany; ^10^ German Center for Child and Adolescent Health (DZKJ), Partner Site Hamburg Hamburg Germany

**Keywords:** bispecific NK‐cell engager (NKCE/BiKE/BiNKE), bispecific T‐cell engager (TCE), EMPD/CεmX, IgE‐associated allergic diseases, immunotherapy

## Abstract

**Background:**

IgE‐associated allergic diseases, affecting approximately 30% of the global population, are a major health burden. Standard treatments, including pharmacotherapy and allergen immunotherapy (AIT), have limitations such as adverse effects, insufficient symptom relief and long treatment durations.

**Methods:**

In this study, we developed and evaluated a bispecific T‐cell engager (TCE) and a half‐life extended (HLE) bispecific natural killer (NK) cell engager (NKCE) targeting IgE‐producing cells, to offer novel, potentially curative therapeutic options. The TCE and HLE‐NKCE constructs were designed to target the CεmX domain of membrane‐bound IgE (mIgE) and engage T cells and NK cells, respectively. Their efficacy was assessed through in vitro luciferase‐based cytotoxicity assays using Ramos and U266 B‐cell lines expressing mIgE as target cells, and primary human immune effector cells and the NK‐cell line NK‐92 as effector cells. Additional characterisation of the constructs was performed using flow cytometry.

**Results:**

Both TCE and HLE‐NKCE specifically mediated killing of CεmX‐expressing cells in a dose‐ and effector‐to‐target (E:T) ratio‐dependent manner. The TCE demonstrated robust activity even at low antigen densities and concentrations as low as 1 ng/mL (18 pM). The HLE‐NKCE effectively mediated killing in the presence of human serum albumin, suggesting functionality in physiological conditions. T‐ and NK‐cell activation was observed only in the presence of target cells and TCE.

**Conclusion:**

Our novel anti‐CεmX TCE and HLE‐NKCE effectively target and eliminate mIgE‐expressing cells in vitro. Although further investigation is needed, our bispecific engagers are promising off‐the‐shelf therapeutics for the treatment of IgE‐associated allergic diseases.

## Graphical abstract

We developed bispecific TCE & NKCE targeting the CεmX domain of mIgE to eliminate IgE‐producing cells. Both constructs demonstrated potent, specific, dose‐dependent cytotoxicity and selective immune activation in vitro. These results highlight their promise as off‐the‐shelf, potentially curative therapeutics for IgE‐associated allergic diseases. Abbreviations: Fc, fragment crystallisable; GzB, granzyme B; mIgE, membrane‐bound IgE; Nb, nanobody; NKCE, natural killer cell engager; scFV, single‐chain variable fragments; TCE, T‐cell engager; VH, variable region of the heavy chain; VL, variable region of the light chain.

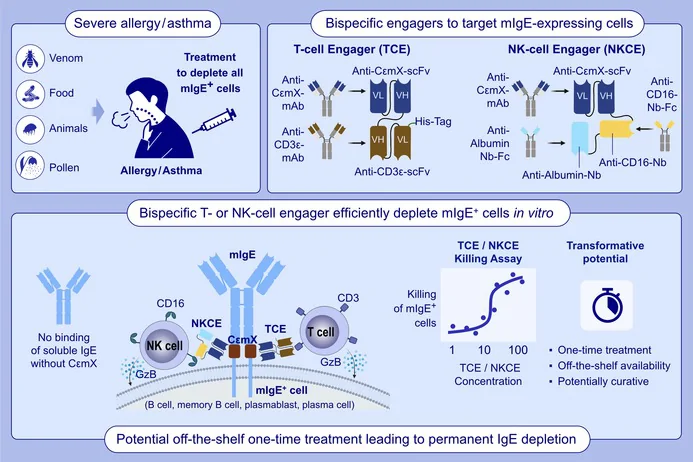

**Abbreviations**
7‐AAD: 7‐Aminoactinomycin D
ABC: antibody binding capacity
ADCC: antibody‐dependent cellular cytotoxicity
AIT: allergen immunotherapy
BC: buffy coat
BCA test: bicinchoninic acid test
BCMA: B‐cell maturation antigen
BCR: B‐cell receptor
BLI: bioluminescence intensity
BSA: bovine serum albumin
CD: cluster of differentiation
cDNA: complementary deoxyribonucleic acid
cps: counts per second
CV: column volume
DMSO: dimethyl sulfoxide
DPBS: Dulbecco's Phosphate‐Buffered Saline

*E. coli*

:
*Escherichia coli*

E:T: effector:target
eGFP: enhanced green fluorescent protein
EMPD: extracellular membrane‐proximal domain
FACS: fluorescence‐activated cell sorting/scanning
Fc: fragment crystallisable
FCS: foetal calf serum
G/Gly: Glycine
hcAbs: heavy chain antibodies
hCD16: human cluster of differentiation 16
HEK 293‐6E: Human Embryonic Kidney 293‐6E (cell line)
His: Histidine
HLE: half‐life extended
HSA: human serum albumin
Ig: immunoglobulin
IL‐4: interleukin 4
Killmax: maximum killing
LLPC: long‐lived plasma cells
luc: luciferase
mAb: monoclonal antibody
MEM: minimum essential medium
MHC: major histocompatibility complex
mIgE: membrane‐bound immunoglobulin E
MW: molecular weight
Nb: nanobody
Ni‐NTA: nickel‐nitrilotriacetic acid
NK cell: natural killer cell
NKCE: (bispecific) natural killer cell engager
NKT cell: natural killer T cell
PBMC: peripheral blood mononuclear cell
PBS: phosphate‐buffered saline
PC: plasma cell
PE: phycoerythrin
PMA: phorbol 12‐myristate 13‐acetate
RPMI: Roswell Park Memorial Institute (medium)
S/Ser: Serine
scFv: single‐chain variable fragment
SDS‐PAGE: sodium dodecyl sulfate–polyacrylamide gel electrophoresis
SEC: size‐exclusion chromatography
sIgE: soluble immunoglobulin E
SLPC: short‐lived plasma cells
TCE: (bispecific) T‐cell engager
VH: variable region of the heavy chain
VL: variable region of the light chain

## 1 Introduction

Allergic diseases are aberrant immune responses to otherwise innocuous molecules, known as allergens. Affecting approximately 30% of the world population, immunoglobulin E (IgE)‐associated allergic diseases, including allergic asthma, allergic rhinitis, allergic dermatitis, allergic conjunctivitis, as well as food and drug allergies [1, 2, 3, 4], are the most prevalent type of allergic diseases with continuously rising incidence [5, 6, 7, 8].

The treatment of IgE‐associated allergies has traditionally focused on avoidance of antigen contact, which is not always feasible. Following allergen exposure, pharmacotherapy is employed to alleviate symptoms [4, 7, 9]. However, traditional pharmacotherapy, which primarily involves H1‐antihistamines, corticosteroids and leukotriene receptor antagonists, requires continued, potentially lifelong administration. Moreover, these therapies are not free of adverse effects, and patients often experience insufficient symptom relief when using pharmacotherapy alone [4, 10].

The only established therapy that can achieve long‐term allergen tolerance is allergen immunotherapy (AIT). However, this potentially curative therapy is not available for all allergens, typically requires three years of treatment to achieve long‐term efficacy, and can lead to adverse reactions [4, 11]. Moreover, potential long‐term side effects of AIT remain to be elucidated.

There have been many efforts to identify new therapeutic options for IgE‐associated allergic diseases. IgE itself has been considered a promising therapeutic target for treating IgE‐mediated allergic diseases, as it plays a central pathophysiological role [12, 13]. This has been underlined by the clinical benefits of anti‐IgE monoclonal antibodies like Omalizumab, which have demonstrated that depletion of IgE can be a safe and effective therapeutic approach [14, 15, 16].

Typically, after loss of tolerance to an otherwise harmless allergen during an initial sensitisation stage, allergen‐specific IgE is produced. This allergen‐specific IgE then binds to Fcε receptors on mast cells, basophils, or eosinophils [17]. Upon re‐exposure, the allergen binds to the specific IgE, crosslinking the IgE‐Fcε complexes and thus leading to the release of inflammatory mediators such as histamine and leukotrienes. This cascade results in early‐phase allergic symptoms that can culminate in anaphylaxis [4, 9].

Physiologically, IgE is involved mainly in immune responses against parasites and is also thought to play a protective role against venoms [1, 18, 19]. However, nowadays in industrial countries, allergies are the main cause of elevated IgE serum levels [20], as parasitic infections have become less common and generally well treatable. Therefore, depleting soluble IgE using Omalizumab is considered to be safe and well tolerated [14, 21, 22]. Whereas Omalizumab has demonstrated the principal feasibility of anti‐IgE therapy, it is not curative but requires regular administration every 2–4 weeks [23]. A potentially curative therapy could work by permanently eliminating IgE production. However, this is complicated by the still uncertain mechanisms of IgE production and the incomplete characterisation of IgE‐producing cells [3, 24, 25, 26, 27, 28, 29]. The current understanding is that IgE is produced by IgE‐producing plasma cells (PC), which can be long‐lived plasma cells (LLPC) or short‐lived plasma cells (SLPC), and themselves differentiate from IgE‐class‐switched B cells [3].

IgE‐class‐switched B cells are believed to express membrane IgE (mIgE) as part of their B‐cell receptor (BCR) complexes [30, 31]. This mIgE is believed to be downregulated upon differentiation into an antibody‐secreting PC [31].

In contrast to their soluble forms, membrane‐bound immunoglobulins of all isotypes have three additional structural features: a cytoplasmic domain, a transmembrane domain and an extracellular membrane‐proximal domain (EMPD). As a result of alternative splicing, mIgE can exist in one of two functional forms of the ε_EMPD_ domain: either the 15 amino acid long ε_short_ or the 67 amino acid long ε_long_, with ε_long_ thought to be predominantly expressed [31, 32]. The EMPD, particularly the unique 52 amino acid long extra domain CεmX only present in ε_long_ [33, 34], is a promising antigenic site to target IgE‐expressing B cells [35]. Several antibodies targeting this domain have been developed [36, 37, 38, 39, 40, 41].

One such antibody, Quilizumab, which targets the CεmX‐containing domain of mIgE, has been clinically explored up to a phase‐II trial for the treatment of allergic asthma [38, 42, 43, 44]. Although Quilizumab was well tolerated, considered safe to be used and able to reduce total as well as allergen‐specific sIgE levels, it showed no clinical benefit in a large cohort of patients with allergic asthma, and its development was therefore discontinued [43, 44].

One potential way to improve the efficacy of mIgE‐targeting therapies is to use alternative platforms besides traditional antibodies, such as bispecific engagers. Bispecific engagers are a type of bispecific antibody and a promising class of immunotherapeutic agents that have gained increasing attention in recent years [45, 46]. These molecules have been designed to simultaneously bind to immune effector cells, such as T cells or natural killer (NK) cells, and to target cells of interest, bringing them into close proximity. Thus, the immune effectors are not only ‘engaged’ but activated to selectively target and eliminate the respective target cell, for example, cancer cells.

The most prominent bispecific engager is Blinatumomab, an anti‐CD3ε–anti‐CD19 bispecific T‐cell engager (TCE), approved for the treatment of adult and paediatric patients with CD19‐positive B‐cell precursor acute lymphoblastic leukaemia [47]. At least two separate research groups have created several analogous TCE constructs that target mIgE [48, 49]. Most of those constructs bind both mIgE and sIgE, which is likely to significantly reduce the efficacy of the mIgE‐targeting treatment. In contrast, Rodak and colleagues developed a CεmX‐targeting engager based on Quilizumab that does not bind to sIgE. However, that engager was unable to activate T cells, and therefore ineffective [49]. Nevertheless, the authors suggested that a similar construct that does not interact with sIgE but elicits potent T‐cell activation could be advantageous for the specific killing of IgE‐producing cells [49].

Here, we present two novel bispecific engagers targeting the CεmX domain of mIgE‐expressing cells: a new TCE and a half‐life extended (HLE) bispecific NK‐cell engager (NKCE). These constructs, while functioning on similar principles, differ in their formats and the immune cells they engage (Figure 1). The TCE binds CD3ε on T cells, having the important advantage of direct T‐cell engagement, in contrast to, for example, full‐length monoclonal antibodies targeting the CεmX‐domain. The HLE‐NKCE, on the other hand, targets CD16 (Fcγ receptor III) on NK cells. Despite CD16 being engaged by traditional IgG antibodies mediating antibody‐dependent cellular cytotoxicity (ADCC), an NKCE that selectively targets CD16 can be expected to be more efficient than a traditional IgG antibody. This is because traditional IgG antibodies not only bind to the activating Fcγ receptor III (CD16) but also engage the inhibitory receptors Fcγ receptor IIB1 and Fcγ receptor IIB2. In contrast, a CD16‐targeting NKCE exclusively engages the activating Fcγ receptor III (CD16), thereby avoiding the dampening of the immune response through binding to inhibitory receptors [50].

**FIGURE 1:**
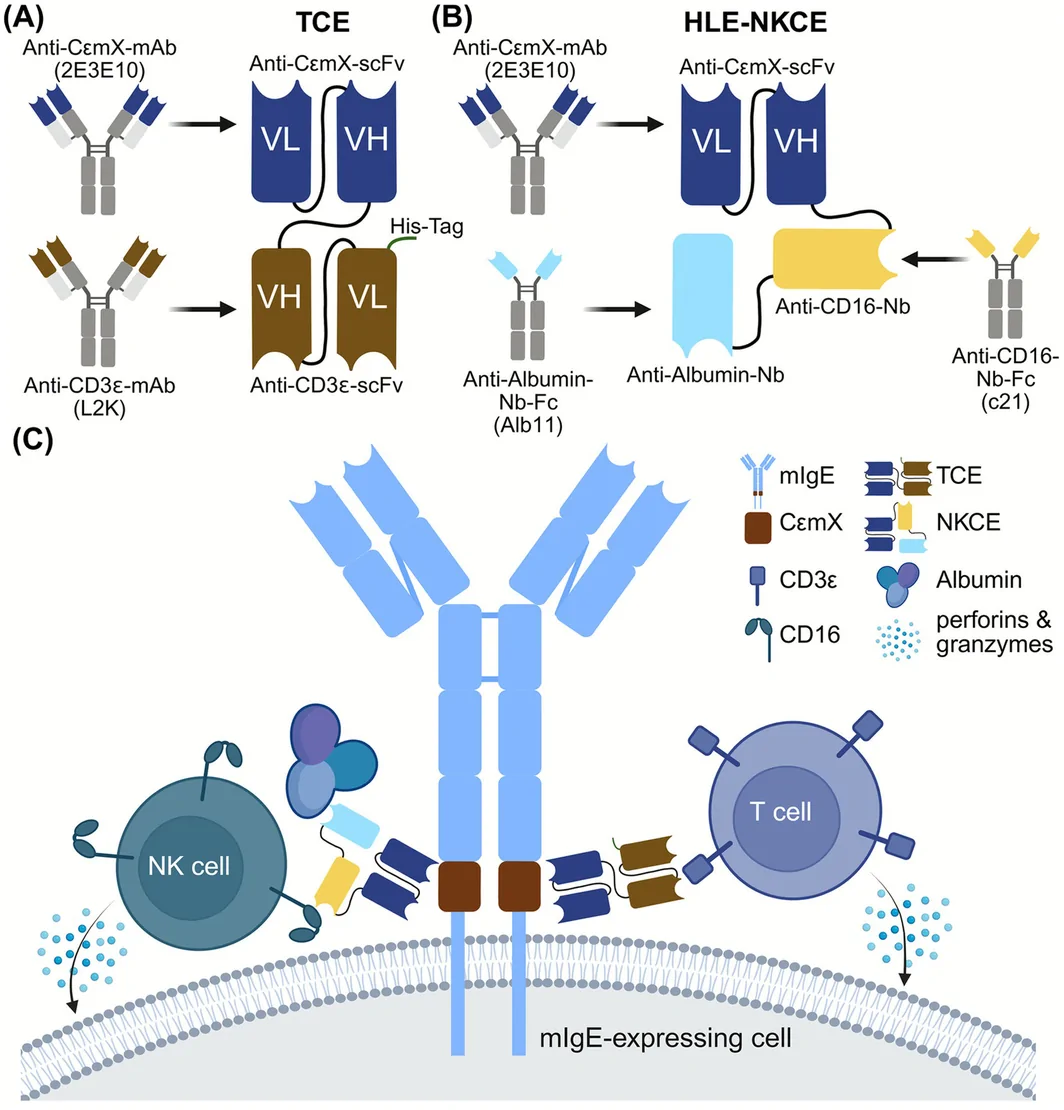
TCE and HLE‐NKCE structure and working mechanisms. (A) Schematic representation of the TCE structure, illustrating the origin and arrangement of its components, including the order of the variable regions of the light chain (VL) and the variable regions of the heavy chain (VH) within the single‐chain variable fragments (scFvs) that target CD3ε and the CεmX domain of mIgE. (B) Schematic representation of the HLE‐NKCE structure, depicting the origin and arrangement of its components, including the order of the VL and VH regions of the CεmX‐targeting scFv, as well as the nanobodies (Nb) against CD16 and human serum albumin (HSA). (C) Conceptual sketch elucidating the working mechanism of the TCE and the HLE‐NKCE. The illustration shows how these bispecific engagers bring effector cells (T cells for TCE and NK cells for HLE‐NKCE) into close proximity with mIgE‐expressing target cells, mediating the killing of the target cell. Binding to albumin (in the case of the HLE‐NKCE) should increase the half‐life of the molecule. Not drawn to scale. Created in BioRender. Möller, C. (2025) https://BioRender.com/hevu2vj.

In our HLE‐NKCE, we used so‐called nanobodies (Nbs), which are highly soluble, antigen‐binding single variable heavy‐chain immunoglobulin domains derived from heavy‐chain antibodies (hcAbs) discovered in camelids [51, 52]. Compared to frequently used single‐chain variable fragments (scFvs), nanobodies offer higher solubility, greater stability, and lower production costs, making them favourable alternatives [53]. Our HLE‐NKCE also includes a third antigen‐binding domain, a nanobody targeting human serum albumin (HSA) with the purpose of extending its otherwise short half‐life (Figure 1B). The NKCE construct is small enough to be filtered by the kidneys; however, by attaching it to HSA, its half‐life might be prolonged to several days, as observed in similar constructs [51, 54]. In addition to avoiding renal filtration due to its large size, HSA binds to the neonatal Fc receptor (FcRn), which protects HSA from lysosomal degradation, further extending its lifespan [55].

Using in vitro luciferase‐based cytotoxicity assays, we demonstrate the effective and specific elimination of target cell lines expressing the CεmX domain of mIgE, even at low antigen densities, mediated by our engager constructs. Furthermore, we examine T‐cell activation as a result of TCE‐mediated T‐cell engagement and explore possible combination therapies of our two constructs together with Omalizumab in vitro.

## 2 Results

### 2.1 TCE & HLE‐NKCE Specifically and Effectively Mediate the Killing of Ramos Cells Expressing the CεmX Domain

Both bispecific engagers, the TCE and the HLE‐NKCE, are designed around a single‐chain variable fragment (scFv) isolated from the conventional antibody 2E3E10, targeting the CεmX domain of mIgE [40]. In the TCE construct, another scFv targeting CD3ε, isolated from the conventional antibody L2K, which has already been used in the clinically successful Blinatumomab [56, 57], is connected to the 2E3E10 scFv via a Gly‐Ser linker [58] (SSGGGGS) (Figure 1A). In the HLE‐NKCE construct, the 2E3E10‐scFv is connected to a CD16‐targeting nanobody [50] through the peptide linker GGGGSGGGS, which is itself connected to a nanobody against HSA [59] via the peptide linker SSGGGGSGGGS (Figure 1B).

Following molecular cloning, production and purification, the integrity and purity of our CεmX‐targeting TCE and HLE‐NKCE were confirmed using SDS‐PAGE (Figure S1A). To assess the functionality of the TCE and HLE‐NKCE, we utilised luciferase‐based cytotoxicity assays [60]. Ramos^luc2/eGFP^ cells expressing the mIgE heavy chain either with (hεmL) or without (hεmS) the targeted CεmX domain (Figure S1B) were used as target and control cells, respectively. For simplicity, we will refer to the Ramos^luc2/eGFP^ hεmL cells as ‘hεmL’ and Ramos^luc2/eGFP^ hεmS cells as ‘hεmS’ from this point forward. For TCE testing, Ramos cells were co‐incubated with primary human T cells at E:T ratios of 10:1 and 5:1. The TCE was added at various concentrations ranging from 0.1 to 1000 ng/mL, corresponding to approximately 1.8 pM to 18 nM. The results demonstrated that the TCE effectively mediated killing of hεmL cells, which express the CεmX domain, while sparing hεmS cells, which lack the CεmX domain. The efficacy of the TCE was time‐ and dose‐dependent, with measurable cytotoxicity at concentrations as low as 1 ng/mL (18 pM) (Figure 2A,B).

**FIGURE 2:**
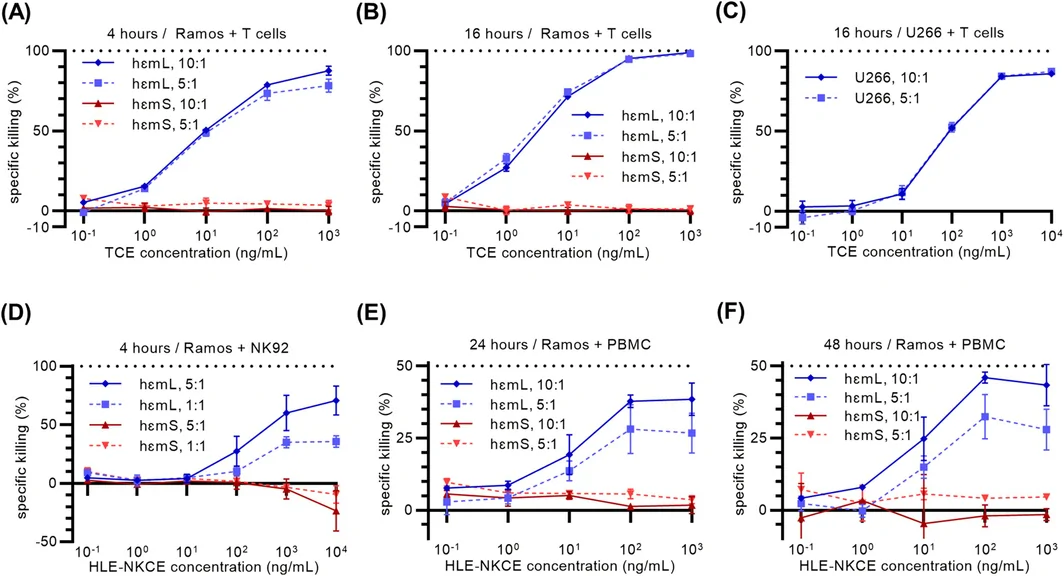
Calculated specific killing activity (%) mediated by the respective bispecific engagers in various cytotoxicity assays. Cytotoxicity assays were repeated with cells from different donors, cell line batches, and bispecific‐engager batches. Ramos^luc2/eGFP^ hεmL (‘hεmL’), depicted in blue, and Ramos^luc2/eGFP^ hεmS (‘hεmS’), depicted in red, were used as target cells in (A), (B), (D–F). U266^luc2/eGFP^, depicted in blue, were used as target cells in (C). Labels are structured as follows: Target cell line, effector‐to‐target ratio (A–D) or PBMC‐to‐target ratio (E, F). Effector cell types and incubation times are indicated. PBMCs and primary human T cells were from different donors. T cells were expanded for four days before use. All experiments were performed in independent triplicates.

The efficacy of the HLE‐NKCE was assessed using analogous luciferase‐based cytotoxicity assays employing NK‐92^hCD16,CD38−/−^ cells, hereafter referred to as only ‘NK92’, and primary human PBMCs as effector cells. Similarly to the TCE, the HLE‐NKCE specifically and effectively mediated the killing of CεmX‐expressing cells in a time‐, dose‐, and E:T ratio‐dependent manner (Figure 2D–F). Compared to the TCE construct, a higher HLE‐NKCE concentration of 10 ng/mL (180 pM) was required to mediate measurable killing. Additionally, the HLE‐NKCE exhibited a slightly lower percentage of calculated specific killing compared to the TCE at comparable E:T ratios, time points and antibody concentration. This difference may be partially explained by the general cytotoxic activity of the NK92 cells even in the absence of the HLE‐NKCE, leading to comparatively high non‐specific killing in the controls (not shown).

In the cytotoxicity assays with primary human PBMCs, PBMC‐to‐target ratios of 10:1 and 5:1 were used. This translated to effective E:T ratios of approximately 2:1 and 1:1, due to the low percentage of CD16+ cells and even lower numbers of NK cells in the PBMC samples. Despite this, the HLE‐NKCE still specifically mediated the killing of hεmL cells, proving that the HLE‐NKCE is effective not only with engineered NK92 cells but also with primary human NK cells and potentially other CD16‐bearing cells (Figure 2E,F).

### 2.2 TCE Mediates the Killing of Low‐Antigen‐Density Cells

After confirming the functionality of the TCE construct, we aimed to test its efficacy on cells with a low antigen density.

The myeloma cell line U266 is known to express mIgE at very low levels [61, 62, 63]. To validate this, we quantified surface IgE expression by determining the antibody binding capacity (ABC). As expected, our engineered Ramos cells expressed high levels of surface IgE, with mean values of 112,000 anti‐IgE antibodies bound per cell for hεmL and 236,000 antibodies per cell for hεmS. In contrast, U266 cells bound on average only 326 anti‐IgE antibodies, confirming the extremely low antigen density. Despite this, the TCE effectively mediated the killing of U266 cells by primary human T cells (Figure 2C). This result is particularly promising, as IgE‐class‐switched B cells are believed to downregulate mIgE upon differentiation into antibody‐secreting plasma cells [31]. In contrast to the promising efficacy of the TCE, cytotoxicity assays on U266 cells using the HLE‐NKCE and NK92 cells as effector cells did not show specific killing activity.

### 2.3 Further Characterisation of the HLE‐NKCE and Its HSA‐Binding Properties

Next, we characterised the HLE‐NKCE, focusing on the HSA‐binding nanobody. We first performed albumin‐binding assays and showed that the HLE‐NKCE can simultaneously bind to human CD16 and HSA (Figure S2A,B). We further tested whether the presence of HSA in vitro affects the killing efficacy of the HLE‐NKCE. Cytotoxicity assays with added HSA revealed that the presence of HSA did not impair the killing efficacy of the HLE‐NKCE in vitro (Figure S2C). This result is encouraging as it suggests that the HLE‐NKCE might function effectively in physiological conditions where HSA is abundant.

### 2.4 Characterisation of the Biophysical Properties of TCE and HLE‐NKCE

Production yields reached up to 3 μg/mL culture supernatant for the TCE and up to 37 μg/mL for the HLE‐NKCE. A two‐step purification protocol, comprising protein A affinity chromatography and size‐exclusion chromatography (SEC), was used to purify the HLE‐NKCE in the analysed production. The final SEC chromatogram displayed three symmetrical peaks, referring to a distribution of particles with three distinct sizes (Figure S3A). As mass spectrometry (data not shown) and SDS‐PAGE (Figure S3C) confirmed the homogeneity of the sample of more than 95%, the SEC data suggest that the three particle sizes refer to three different oligomeric states. The occurrence of three oligomeric states of HLE‐NKCE was mirrored in the mass photometer results (Figure S3B), where Peak A eluted primarily trimers (~188 kDa), Peak B eluted primarily dimers (~122 kDa) and Peak C eluted primarily monomers (~59 kDa).

By contrast, the analysed small‐scale TCE production yielded only limited material, which hampered purification and resulted in a heterogeneous final sample. The elution peak from the final SEC step appeared asymmetrical, with larger molecular weight contaminations occurring (Figure S4A). Mass photometer and SDS‐PAGE analyses also indicated sample heterogeneity (Figure S4B,C). Other TCE batches did not show such pronounced heterogeneity in SDS‐PAGE (Figure S1A), likely because of better purification due to relatively higher TCE amounts. Given the limited amount of TCE in the analysed batch and the detection limits of our assays, we cannot unambiguously distinguish whether the observed high‐molecular‐weight signal arises from TCE oligomerisation or from co‐eluting contaminants. However, a tendency of the TCE to aggregate may be expected, as bispecifics comprising two scFvs have been shown to be prone to aggregation [64].

### 2.5 TCE & HLE‐NKCE Induce Robust Effector Cell Activation Only in the Presence of Target Cells

To visualise effector‐cell activation, we conducted co‐culture experiments using PBMCs and analysed the results via flow cytometry. Our findings revealed that the T‐cell‐activation markers CD25 and CD69 were highly expressed on both CD4^+^ and CD8^+^ immune cells when co‐cultured together with 400 ng/mL (7 nM) TCE and hεmL target cells. In contrast, only slight expression of these markers could be observed when PBMCs were incubated with TCE or target cells alone (Figure 3). The level of activation, as indicated by the expression of activation markers, was stronger than that induced by the T‐cell stimulating reagent TransAct. These illustrative results demonstrate that the TCE induces polyclonal and major‐histocompatibility‐complex (MHC‐) independent T‐cell activation of both CD4^+^ and CD8^+^ T cells only when both TCE and target cells are present. In a similar flow cytometry assay measuring the activation marker CD69 and the degranulation marker CD107a on CD3^−^, CD56^+^ PBMCs, strong upregulation of both markers was observed when both HLE‐NKCE and target cells were present. In this illustrative experiment, HLE‐NKCE in the absence of target cells resulted in a modest elevation of CD69 and CD107a compared to unstimulated controls (Figure S5). This slight elevation could be explained by CD16 cross‐linking by HLE‐NKCE multimers. Importantly, this suspected NK‐cell activation did not manifest as functional cytotoxicity, as no specific killing activity on antigen‐negative cells was detected across the tested HLE‐NKCE concentration range (Figure 1D,E).

**FIGURE 3:**
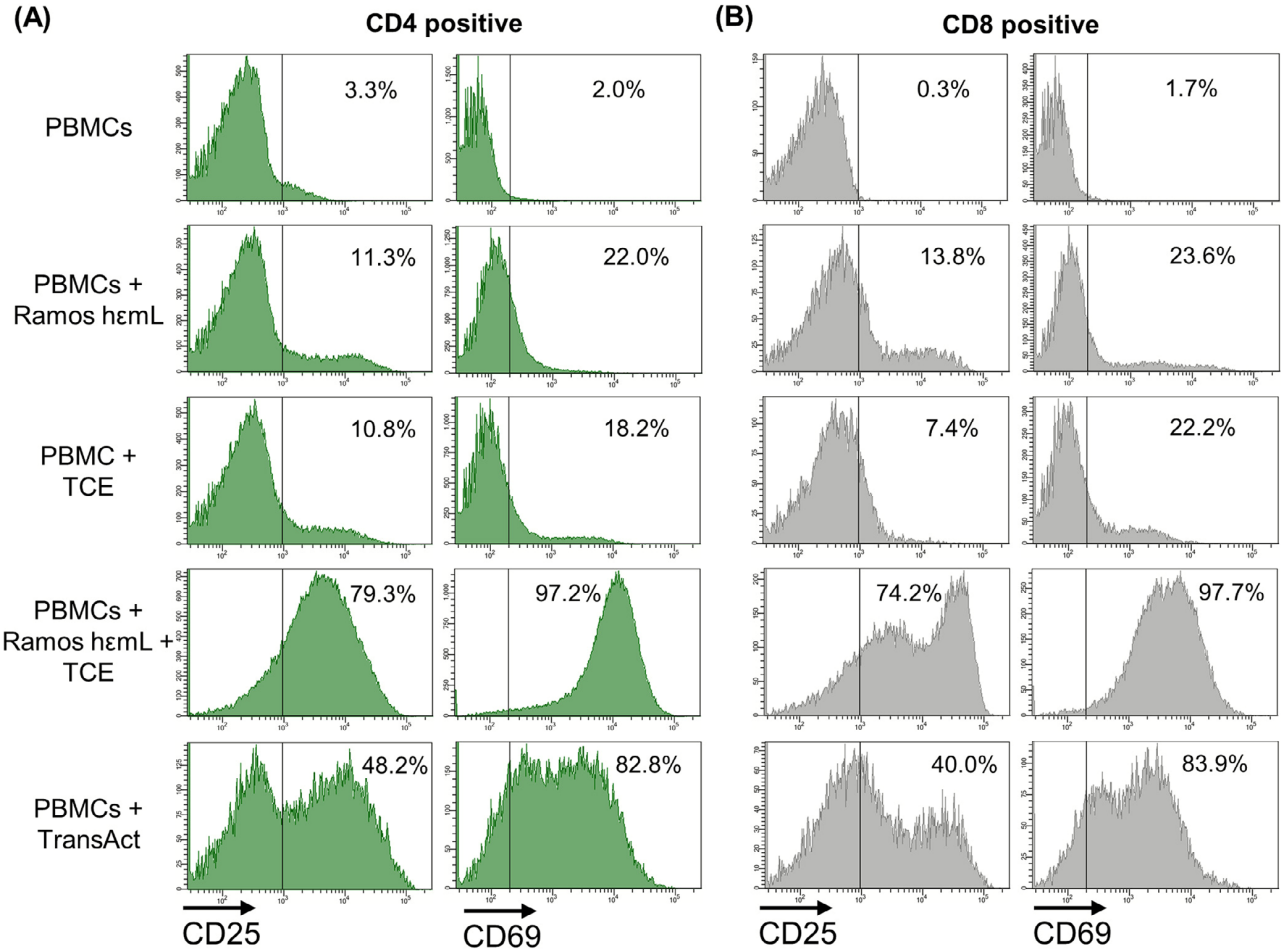
Illustrative flow cytometry analysis of T‐cell activation. Histograms showing the CD25 and CD69 expression levels in CD4^+^ (A) and CD8^+^ (B) immune‐effector subsets. PBMCs were incubated for 2 days under various conditions and then stained and analysed using flow cytometry.

### 2.6 Comparative Analysis and Characterisation of TCE, HLE‐NKCE and Omalizumab

In the final set of experiments, we evaluated the efficacy of our constructs in comparison to and in combination with Omalizumab. First, we performed four‐hour cytotoxicity assays utilising NK92 cells and found a superior ability of the HLE‐NKCE to mediate killing of hεmL cells compared to Omalizumab. Notably, in the case of co‐application, the presence of Omalizumab did not affect the ability of the HLE‐NKCE to mediate killing (Figure 4A).

**FIGURE 4:**
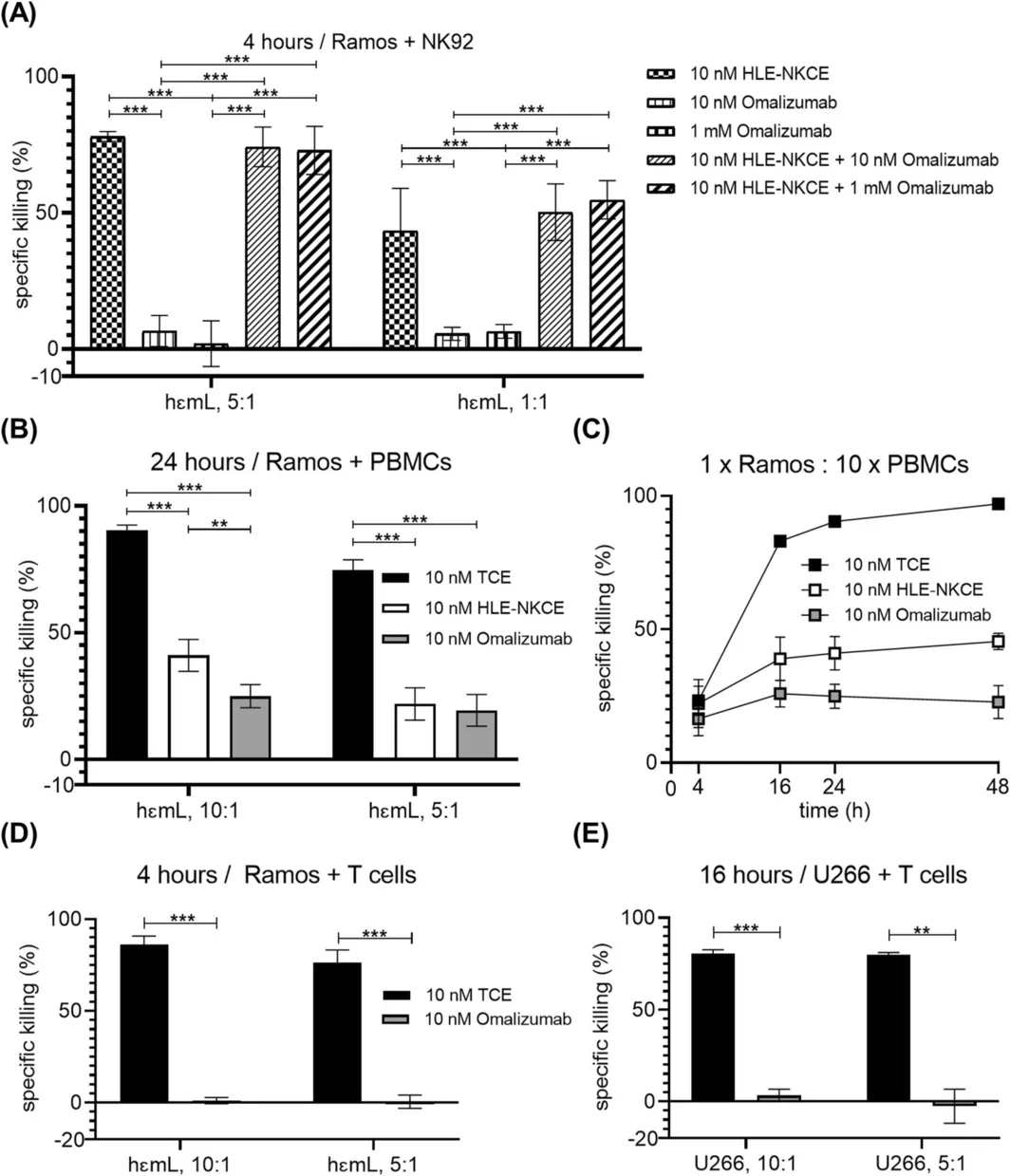
Calculated specific killing activity (%) mediated by the respective bispecific engagers and Omalizumab in various cytotoxicity assays. Cytotoxicity assays were repeated with cells from different donors, cell line batches, or bispecific engager batches. Ramos^luc2/eGFP^ hεmL (‘hεmL’) (A–D) and U266^luc2/eGFP^ (‘U266’) (E) were used as target cells. Value labels depict the antibody concentrations. *X*‐axis labels for (A), (B), (D), and (E) are structured as follows: Target cell line, effector‐to‐target ratio (A, D, E) or PBMC‐to‐target ratio (B). The legend of panel (D) also applies to panel (E). Experiments depicted in (A) were performed in independent duplicates, (B–E) in independent triplicates. The displayed *p*‐values in (A) + (B) were calculated using Tukey's multiple comparisons test following a two‐way ANOVA. The *p*‐values in (D,E) were calculated using multiple *t*‐tests. A *p*‐value < 0.05 is deemed significant (*), a *p*‐value < 0.01 highly significant (**), and a *p*‐value < 0.001 very highly significant (***). Insignificant *p*‐values of ≥ 0.05 are not displayed.

Next, we tested our constructs and Omalizumab, all at the same molar concentration of 10 nM, corresponding to approximately 558 ng/mL TCE, 570 ng/mL HLE‐NKCE and 1490 ng/mL Omalizumab, using unstimulated PBMCs as effector cells in 24‐h cytotoxicity assays. The results showed that the TCE‐mediated killing was most efficient (Figure 4B,C). This is likely due to the higher number of T cells compared to NK cells in the PBMC samples (data not shown) and a higher efficacy of the TCE compared to the HLE‐NKCE at low concentrations. The HLE‐NKCE still exhibited higher killing compared to Omalizumab, although the difference was less pronounced than in the previously mentioned cytotoxicity assay with NK92 cells (Figure 4A). Interestingly, Omalizumab showed strong killing of hεmS cells compared to hεmL cells (Figure S6A). This may reflect more efficient ADCC, for example, because Omalizumab binds closer to the cell membrane on hεmS cells than on hεmL cells [65], or simply because of the higher antigen density on hεmS. Another explanation could be mIgE crosslinking‐induced apoptosis, previously found to occur nearly exclusively with short mIgE lacking the CεmX domain [37, 66, 67]. However, this mechanism remains controversial, specifically with regard to Omalizumab [68].

In cytotoxicity assays with primary human T cells, the TCE mediated significantly superior killing of hεmL and U266 cells compared to Omalizumab (Figure 4D,E). In these assays, we observed an even greater difference in the cytotoxic activity of Omalizumab on hεmS cells compared to hεmL cells (Figure S6B). Given that the level of cytotoxic activity observed is unlikely to be attributed solely to the cytotoxic activity of the small CD16^+^ T‐cell subset, including, for example, natural killer T (NKT) cells, this finding points towards an ADCC‐independent mechanism of Omalizumab activity. Indeed, when target cells were incubated with Omalizumab alone, we observed measurable cytotoxic activity (Figure S6C). This could be explained by mIgE crosslinking inducing apoptosis. Differences between hεmL, hεmS, and U266 cells might be attributed to their different antigen densities or to a higher susceptibility of the short mIgE isoform for apoptosis.

In summary, our results demonstrate a remarkable efficacy of the novel TCE and HLE‐NKCE constructs in mediating specific and effective killing of CεmX‐expressing target cells in vitro. Both constructs mediate time‐, dose‐, and E:T‐ratio‐dependent cytotoxicity, with the TCE exhibiting particularly robust activity even at very low antigen densities and at TCE concentrations as low as 1 ng/mL (18 pM).

## 3 Discussion

Given the very high and still continuously rising global incidence of IgE‐associated allergic diseases as well as the often limited treatment options, there is an urgent need for novel therapeutic strategies. In this study, we developed and tested two novel bispecific engagers, a TCE and an HLE‐NKCE, both targeting the CεmX domain of mIgE‐expressing cells as potential new off‐the‐shelf therapeutics, particularly for the treatment of severe diseases.

Previous work by Rodak et al. [49] described the creation of a CεmX‐targeting TCE based on Quilizumab, an antibody targeting the mIgE fragment containing CεmX. However, their Quilizumab‐TCE was unable to activate T cells, rendering it ineffective [49]. In contrast, our CεmX‐targeting TCE demonstrated robust and specific cytotoxic activity against CεmX‐expressing cells, including those with low antigen density. At the same time, we showed that our TCE induces robust T‐cell activation only in the presence of antigen‐expressing target cells. This observation is particularly encouraging, since (unspecific) T‐cell activation has been linked to the majority of adverse events in TCE therapy [45, 69, 70]. Two additional factors, namely the expectedly very low abundance of IgE‐producing cells [26] and the unlikeliness of off‐target binding of the used CεmX‐targeting scFv, could be expected to further contribute to a much lower probability of unspecific, broad T‐cell activation and resulting adverse events for our TCE as compared to clinically used TCE therapies targeting much more abundant cancer cells and antigens. Notably, the situation might be different if a CεmX‐targeting TCE were to be used in the context of (very rare) IgE‐positive multiple myeloma [71].

To our knowledge, the HLE‐NKCE presented here is the first described NKCE targeting CεmX or even mIgE. It effectively and specifically mediated the killing of target cells with high antigen density in a dose‐ and E:T‐ratio‐dependent manner but showed no observable cytotoxic activity on U266 cells known to have low antigen density. We designed our NKCE with a nanobody against albumin to prolong serum half‐life. We did not observe a negative impact of HSA binding on the efficacy of the HLE‐NKCE, demonstrating that the construct should function effectively in physiological conditions where HSA is abundant. In a future therapeutic regimen, a half‐life extension may reduce the frequency of dosing or eliminate the need for continuous administration, but it may also result in less therapeutic control, since an HLE‐construct would be expected to stay in the body for days, even after stopping its administration. Although the probability of adverse effects of CεmX‐targeting constructs is currently considered to be low, mainly because of their expectedly high specificity in combination with the low abundance of target cells (s. above), the reduced manageability of HLE‐bispecific engagers would have to be taken into account in the context of clinical applications.

Although most IgE is thought to be produced by short‐lived IgE B and plasma cells [28], it is becoming increasingly clear that LLPCs are also an important source of IgE [43, 44, 72]. Therefore, an effective therapeutic strategy would likely need to target IgE LLPCs as well. Quilizumab, an mIgE‐targeting conventional antibody, failed to eliminate LLPCs, likely due to the low to possibly non‐existent expression of mIgE on these cells [43, 44]. The actual mIgE expression level on IgE LLPCs remains unknown [26, 28, 29], making it theoretically possible that mIgE‐targeting therapeutics effective at low antigen densities, like our TCE, might be able to deplete IgE LLPCs.

A potential combination partner for our constructs would be a B‐cell maturation antigen (BCMA)xCD3 bispecific antibody, which can kill PCs including IgE PCs [72]. However, treatment with the BCMAxCD3 bispecific antibody presumably spares class‐switched IgE B cells, including potential memory B cells, which could in turn be targetable using our constructs. A combination therapy consisting of the BCMAxCD3 bispecific antibody and our TCE or HLE‐NKCE could theoretically eliminate all IgE‐producing cells. Subsequent continuous IL‐4 receptor α blockade, for example, with Dupilumab [73], has been proposed as an option to prevent novel class switches of allergen‐specific memory B cells to IgE [72], but potential long‐term toxicity needs to be considered [74].

While our study presents promising results showcasing the potential of two novel bispecific engagers targeting the CεmX domain of mIgE‐expressing cells, several limitations need to be acknowledged. An obvious constraint is that our proof‐of‐concept study was conducted in vitro only. Additionally, although our constructs were tested on cell lines with significantly different antigen densities, these cell lines may not accurately represent the state of mIgE‐expressing cells in allergic patients. As it is notoriously difficult to identify or isolate these IgE‐positive cells from patients, animal studies using models such as humanised mice [41] or recently evolved primates [35] would be a reasonable next step to more closely mirror the complexity of the in vivo situation.

In conclusion, our study introduces two novel bispecific engagers that specifically target mIgE. We could show that both engagers mediate highly efficient and highly specific killing of mIgE‐expressing cells; in the case of the TCE, even at low antigen densities. We therefore suggest that these new bispecific engagers warrant further investigation as interesting candidates for off‐the‐shelf therapeutics to treat IgE‐associated allergic diseases.

## Author Contributions

C.M.: conceptualisation, methodology, validation, formal analysis, investigation, writing – original draft, writing – review and editing, visualisation. J.H.: conceptualisation, methodology, resources, writing – review and editing. A.Z.S.: conceptualisation, methodology, resources. T.E.: conceptualisation, methodology, resources. S.C.: methodology, resources. AJG: investigation, methodology. N.T.: investigation, methodology. R.M.‐R: investigation, methodology. P.L.: investigation, methodology. S.Q.: investigation, methodology, writing review and editing. S.W.: investigation, methodology, writing – review and editing. M.E.: investigation, methodology. N.M.T.: investigation, methodology. F.H.: resources. F.K.‐N.: supervision, resources. B.F.: conceptualisation, supervision, funding acquisition, project administration, writing – review and editing. K.R: conceptualisation, methodology, resources, investigation, supervision, project administration, writing – review and editing. All authors read the final version of the manuscript and agreed on its submission.

## Funding

C.M. and S.C. were financially supported by the Else Kröner‐Fresenius‐Stiftung iPRIME Scholarship (2021_EKPK.10), University Medical Center Hamburg‐Eppendorf. K.R. and B.F. were funded by the Deutsche Forschungsgemeinschaft (DFG, German Research Foundation) ‐ SFB 1648‐1 2024 – 512741711. M.E. was supported by the Else Kröner‐Fresenius Foundation as part of the iPRIME‐CS program. N.M.T. was funded by the European Research Council (AUTO‐TARGET) and by the Else Kröner‐Fresenius Foundation (Clinician Scientist Professorship).

## Conflicts of Interest

Authors C.M., J.H., A.Z.S., T.E., F.K.‐N., B.F., K.R. have filed a patent application (EP25157230.1) related to the constructs presented in this manuscript. All other authors have no conflicts of interest within the scope of the submitted work.

## Supporting information


**Appendix S1:** all70236‐sup‐0001‐AppendixS1.docx.

## Data Availability

The data that support the findings of this study are available from the corresponding author upon reasonable request.
