# Supplementary material for: Novel Bispecific Engagers Targeting the CεmX Domain of mIgE‐Expressing Cells
Source: Allergy. 2026 Feb 2;81(11):3853–63. doi: 10.1111/all.70236 (PMC13753656; doi:10.1111/all.70236)
Supplement: Supplementary file 1 [file ALL-81-3853-s001.docx]

**Supplementary Materials**

**Title**

Novel Bispecific Engagers Targeting the CεmX Domain of mIgE-Expressing Cells

**Authors**

Constantin Möller, Julia Hambach, Alessa Zoe Schaffrath, Thomas Eden, Sophia Cichutek, Anna Josephine Gebhardt, Natalie Tode, Ronald Maitschke-Rajasekharan, Philipp Lewe, Sharon Qi, Susanne Witt, Marie Eggers, Nicola M. Tomas, Friedrich Haag, Friedrich Koch-Nolte, Boris Fehse, Kristoffer Riecken

**Content**

- Suppl. Figure S1: Purity analysis of bispecifics and assessment of CεmX expression on Ramos target cells.
- Suppl. Figure S2: HLE-NKCE binding characteristics and evaluation of HSA impact on NK-cell cytotoxicity.
- Suppl. Figure S3: Characterisation of purified HLE-NKCE from a single production batch.
- Suppl. Figure S4: Characterisation of purified TCE from a single production batch.
- Suppl. Figure S5: Illustrative flow cytometry analysis of NK-cell activation and degranulation.
- Suppl. Figure S6: Calculated specific killing activity (%) mediated by Omalizumab in various cytotoxicity assays.
- Material and Methods

**(B)**

**(A)**

Ramos hεmS

Ramos hεmS

CεmX

CεmX

TCE

Marker 1x

Marker 4x

HLE-NKCE

Marker 4x

SSC-A

-50 kDa

-50 kDa

-25 kDa

-66 kDa

-66 kDa

-25 kDa

IgE

FSC-A

Ramos hεmL

Ramos hεmL

-14 kDa

-14 kDa

SSC-A

IgE

FSC-A

**Supplemental Figure S1:** Purity analysis of bispecifics and assessment of CεmX expression on Ramos target cells.

**(A)** Purity of the TCE and HLE-NKCE was assessed using SDS-PAGE followed by Coomassie staining. **(B)** Flow cytometry analysis of Ramos hεmS and Ramos hεmL cells stained with IgE-specific and CεmX-specific (2E3E10) antibodies.

**(A)**

10 ng/mL HLE-NKCE

1 ng/mL HLE-NKCE

0.1 ng/mL HLE-NKCE

10 000 ng/mL HLE-NKCE

1 000 ng/mL HLE-NKCE

100 ng/mL HLE-NKCE

**(B)**

**(C)**

**Supplemental Figure S2:** HLE-NKCE binding characteristics and evaluation of HSA impact on NK-cell cytotoxicity**.**

**(A)** Histograms of HLE-NKCE binding assays. NK92^hCD16; CD38-/-^ cells were incubated with HLE-NKCE at various concentrations, a biotinylated human albumin and stained with a Biotin-PE antibody.

**(B)** Line chart showing the percentage of PE-positive single cells from the same experiment described in panel A.

**(C)** Graph depicting the results of a cytotoxicity assay to test the potential interference of HSA in the function of HLE-NKCE. NK92^hCD16;CD38-/-^ cells were co-incubated with Ramos^luc2/eGFP^ hεmL cells at E:T ratios of 5:1 or 1:1 for 4 hours, either without HSA or with HSA at a final concentration of 4.45 g/L added to the medium.

**(A)**

A

B

C

**(B)**

**(C)**

M	1	2	3	4	5	6	7	8

1.B6

1.B4

1.B2

1.C1

1.C4

1.C5

1.C7

1.C9

kDa

Peak A

75 -

50 -

37 -

150 -

250 -

25 -

20 -

15 -

10 -

100 -

Peak B

Peak C

**Supplemental Figure S3:** Characterisation of purified HLE-NKCE from a single production batch.

**(A)** Chromatogram from automated SEC run on Cytiva ÄKTA pure system a HiLoad 16/600 Superdex 200 pg column. The x-axis represents the elution volume (mL) and corresponding fraction names, the y-axis represents the absorbance at 280 nm. The target protein began elution at ~363 mL in 3 separate peaks (A, B, C).

**(B)** Peaks A, B and C run on a mass photometer shows the monomer molecular weight (MW) of ~55.82 kDa in addition to the multimerization of HLE-NKCE

**(C)** SDS gel loaded with denatured elution fractions shows a uniform singular band at ~55 kDa.

**(A)**

**(C)**

**(B)**

250 -

75 -

50 -

150 -

37 -

25 -

20 -

15 -

10 -

100 -

M	1	2	3	4	5	6	7	8

1.B7

1.B6

1.B5

1.B4

kDa

**Supplemental Figure S4:** Characterisation of purified TCE from a single production batch.

**(A)** Chromatogram from automated SEC run on Cytiva ÄKTA pure system using a Superdex 200 Increase 10/300 column. The x-axis represents the elution volume (mL) and corresponding fraction names, the y-axis represents the absorbance in arbitray units at 280 nm. The target protein eluted at ~75 mL.

**(B)** TCE run on mass photometer shows the monomer MW of ~56.95 kDa.

**(C)** SDS gel loaded with denatured elution fractions shows elution of TCE from ~75 mL elution volume, in addition to contaminating bands at ~50 kDa and ~27 kDa.

PBMC +

NKCE

PBMCs +

Ramos hεmL

PBMCs +

Ramos hεmL +

NKCE

PBMCs

PBMCs +

PMA +

Ionomycin

CD69

CD107a

20.2%

10.4%

99.4%

94.3%

26.8%

13.4%

29.4%

22.0%

83.6%

81.5%

**Supplemental Figure S5:** Illustrative flow cytometry analysis of NK-cell activation and degranulation.

Histograms showing the CD69 and CD107a expression levels on CD3⁻, CD56^+^ PBMCs. PBMCs were incubated for 12 hours under various conditions and then stained and analysed using flow cytometry.

**(A)**

**(B)**

**(C)**

**Supplemental Figure S6:** Calculated specific killing activity (%) mediated by Omalizumab in various cytotoxicity assays. **(A)** and **(B)** show cytotoxicity assays with effector cells. **(C)** shows the effect of Omalizumab alone, without effector cells present, after 24 hours. Cytotoxicity assays were repeated with cells from different donors or cell line batches. Ramos^luc2/eGFP^ hεmL (“hεmL”) and Ramos^luc2/eGFP^ hεmS (“hεmS”) were used as target cells. In **(A)** value labels depict the antibody concentrations (10 nM ≙ 1490 ng/mL Omalizumab) and the respective target cells. In **(B)** value labels depict the antibody concentration while X-axis labels are structured as follows: target cell line, effector-to-target. In **(C)** values depict the target cell line. The x-axis shows concentrations ranging from 1 ng/mL (6.7 pM) to 1 mg/mL (6.7 µM). Experiments were performed in independent triplicates.

**Materials and Methods**

Production of TCE and HLE-NKCE Constructs

The coding sequence of the anti-CεmX scFv 2E3E10 was obtained from the patent US20180022826A1. A codon-optimised version of the complementary DNA (cDNA) was synthesised *de novo*, with VL and VH domains connected by a peptide linker with the amino acid sequence GSTSGSGKPGSGEGS. The coding sequence of the anti-CD3ε-scFv derived from the murine mAb L2K (Micromet)^1^, which is also used in Blinatumomab, was obtained from drugbank.com^2^, codon-optimised and *de novo* synthesised. VH and VL of the anti-CD3ε-scFv are connected by a linker with the amino acid sequence SSVEGGSGGSGGSGGSGGV. For the production of the HLE-NKCE, the 2E3E10-scFv was cloned into the pCSE2.5-WF211-HLE-NKCE expression plasmid^3^ replacing the WF211 nanobody (NcoI/NotI). The expression plasmid already included the CD16-specific nanobody c21 [ref^4^] and the HSA-specific nanobody Alb11 [ref^5^]. For the production of the TCE, the CD16-specific nanobody and the albumin-specific nanobody downstream of the 2E3E10-scFv were replaced by the anti-CD3ε-scFv and a His-tag (NotI/XbaI). In both constructs, the antibodies or nanobodies were connected via Glycine-Serine linkers, for the TCE: 2E3E10-(SSGGGGS)-LK2, and for the HLE-NKCE: 2E3E10-(GGGGSGGGS)-c21-(SSGGGGSGGGS)-Alb11. All cloning steps were verified by test digestions and Sanger sequencing. Detailed cloning information is available upon reasonable request.

The resulting plasmids were used to transiently transfect HEK 293-6E or MEXi293-E cells as previously described^3,6,7^. Both cell lines are derived from HEK 293 cells and express versions of the EBNA-1 protein, which promotes episomal replication of oriP-harbouring plasmids, thereby boosting the respective expression. Supernatants were collected 6 to 8 days post-transfection. The resulting antibody constructs were purified by affinity chromatography, utilising HIS-Select Nickel Affinity Gel (Sigma, St. Louis, MO, USA) for the TCE and Protein A sepharose (GE Healthcare, Düsseldorf, Germany) for the HLE-NKCE. Dulbecco's Phosphate-Buffered Saline without calcium and magnesium (DPBS⁻^/^⁻) (Gibco/ThermoFisher Scientific, Schwerte, Germany) was used as washing and storage buffer. For His-tag affinity purification, proteins were eluted with DPBS⁻^/^⁻ supplemented with 250 mM imidazole (ThermoFisher Scientific). Protein A eluates were obtained using IgG Elution Buffer (ThermoFisher Scientific) and immediately neutralized with Tris Buffer (G-Biosciences, St. Louis, MO, USA).

Desalting was performed using PD-10 columns (GE Healthcare) and the resulting constructs were subsequently concentrated using Amicon Ultra centrifugal filter units (Merck Millipore/SigmaAldrich, St. Louis, MO, USA) with a molecular weight cut-off of 30 kDa. Constructs were eluted and stored in DPBS containing calcium and magnesium (Gibco/ThermoFisher Scientific) at 4°C.

The purity of the produced antibody was assessed by SDS-PAGE followed by staining with InstantBlue Coomassie Protein Stain (Abcam, Cambridge, MA, USA). The expected molecular weights were approximately 57.0 kDa for the TCE and 55.8 kDa for the HLE-NKCE. Protein concentration was determined using a BCA test (Pierce BCA Protein Assay Kit, ThermoFisher Scientific).

Characterisation of the biophysical properties of TCE and HLE-NKCE

For biophysical characterisation, TCE and HLE-NKCE were produced and purified as described above. Automated size-exclusion chromatography (SEC) was performed on an ÄKTA pure system (Cytiva, Marlborough, MA, USA). TCE samples were analysed on a Superdex 200 Increase 10/300 GL column (Cytiva) at a flow rate of 0.75 mL/min and HLE-NKCE samples on a HiLoad 16/600 Superdex 200 pg column (Cytiva) at 1 mL/min. Each column was equilibrated with 1.5 column volumes (CV) of H_2_O followed by 1.5 CV of phosphate-buffered saline (PBS, Invitrogen, Karlsbad, CA, USA/ThermoFisher Scientific) prior to injection. Samples, prepared in PBS, were applied by loop injection and eluted in 1-mL fractions collected over an elution volume of 1.5 CV. Individual peaks were pooled for downstream analyses. Collected SEC fractions were assessed by SDS-PAGE.

Mass photometry was performed on a TwoMP mass photometer (Refeyn, Oxford, UK), calibrated with MassReference P1 (Refeyn). Data were acquired with AcquireMP (Refeyn) and analysed with DiscoverMP (Refeyn). Samples were prepared at 10-20 nM in PBS and applied to the instrument at a 1:18 dilution (sample:buffer). Each sample was measured in two independent replicates.

Cell Source and Ethical Approval

Anonymised buffy coats (BCs) were kindly provided by the local Institute of Transfusion Medicine. The anonymous donors provided written informed consent for the anonymised use of leftover whole-blood donations for research purposes. The local ethics committee of the Chamber of Physicians in Hamburg specifically waived the requirement for ethics approval for the anonymised generation of primary human T cells from otherwise discarded material (Ref-No.: 2023-300348-WF).

Isolation, Storage and Culture of Peripheral Blood Mononuclear Cells (PBMCs)

PBMCs were isolated from fresh BCs and subsequently cryopreserved in foetal calf serum (FCS; Sigma) supplemented with 10% dimethyl sulfoxide, as previously described^8^. Upon thawing, PBMCs were cultured in RPMI culture medium at a cell density of around 2 x 10^6^ cells/mL and incubated at 37°C with 5% CO_2_ and 95% relative humidity. RPMI culture medium consisted of RPMI-1640 (Gibco) supplemented with 10% FCS, 100 U/mL penicillin, and 100 μg/mL streptomycin (Gibco).

T-Cell Culture

To obtain T cells, PBMCs were resuspended in T-cell medium at a cell density of 2 x 10^6^ cells/mL following the thawing steps described above. T-cell medium consisted of TexMACS medium (Miltenyi Biotec, Bergisch Gladbach, Germany) supplemented with 1 mM sodium pyruvate (Gibco), 0.05 mM 2-mercaptoethanol (Gibco), 3.5% human serum (from buffy coats), 20 U/mL IL-2 (Miltenyi Biotec)**,** 100 U/mL penicillin and 100 μg/mL streptomycin. The cells were then incubated at 37°C with 5% CO_2_ and 95% relative humidity. PBMCs were expanded using human T cell TransAct (Miltenyi Biotec) according to the manufacturer's instructions for 4 days. The T-cell medium was changed every 2 days, and cells were split as necessary to maintain a cell density of approximately 2 x 10^6^ cells/mL.

Target Cell and NK-Cell Culture

Cells from the human Burkitt’s lymphoma cell line Ramos, stably expressing firefly luciferase (luc2), enhanced green fluorescent protein (eGFP)^9^ and the heavy chain of either the short version of mIgE, containing ε_short_ (Ramos^luc2/eGFP^ hεmS) or the long version of mIgE containing ε_long_ and therefore the CεmX domain (Ramos^luc2/eGFP^ hεmL), were used as target cells^10^. Ramos cells were cultured in suspension cell culture flasks using RPMI culture medium (s. above), which was changed every 2-3 days. The cells were split as necessary to maintain a cell density of 0.3-1.0 x 10^6^ cells/mL.

The U266 cell line, a B-lymphocyte cell line expressing mIgE at a very low antigen density^11,12^ and also secreting IgE^13^, was transduced to stably express luc2 and eGFP^10^ as re-confirmed by flow cytometry using a NovoCyte Quanteon (Agilent, Santa Clara, CA, USA). The resulting cells were cultured in suspension cell culture flasks using RPMI culture medium. The medium was exchanged every 2-3 days. U266 cells were split as needed to maintain a cell density between 0.3-1.0 x 10^6^ cells/mL.

NK-92 cells stably expressing human CD16 and with a knock-out of CD38 [ref^3^] were used as effector cells. These NK-92^hCD16;CD38-/-^ cells were cultured in suspension cell culture flasks using NK-cell medium. NK-cell medium consisted of Minimum Essential Medium (MEM; Lonza, Verviers, Belgium) α supplemented with 12.5% horse serum (Gibco), 12.5% FCS, 2 mM glutamine (Gibco), 100 U/mL penicillin, 100 μg/mL streptomycin, 0.1 mM 2-mercaptoethanol, and 100 U/mL IL-2. They were maintained at a cell density of 2-5 x 10^5^ cells/mL and cell aggregates were dispersed every 2-3 days by pipetting up and down.

Luciferase-based Cytotoxicity Assay

Luciferase-expressing target cells were suspended in 50 µL of the respective effector cell medium at a cell density of 2 x 10^5^ cells/mL and pipetted into a white 96-well plate (OptiPlate; PerkinElmer, Boston, MA, USA). A 10-µL mixture of the antibody construct diluted in DPBS was then added to the wells, with each condition tested in triplicate. Wells containing 10 µL of DPBS without the antibody construct served as a control. Independent biological repeats were performed as stated in the figure legends.

Five minutes after the addition of the antibody constructs, effector cells suspended in 50 µL of their respective effector cell medium at varying concentrations to ensure different effector-to-target (E:T) ratios were added to the wells (t = 0 h).

The plate was then incubated at 37°C with 5% CO_2_ for 4, 16, 24, or 48 hours, depending on the specific experiment. Twenty minutes before the desired readout, 50 µL of the respective effector cell medium containing 150 µg/mL D-luciferin (Biosynth AG, Staad, Switzerland) were added to each well. Maximum killing (Killmax) was achieved by lysing the target cells with 1% Triton X-100 shortly before adding the D-luciferin containing medium.

After an additional 20-minute incubation, the bioluminescence intensity (BLI) of the luciferase-expressing cells was measured using a bioluminescence reader (Infinite 200 PRO; Tecan, Männedorf, Switzerland) and expressed as counts per second (cps).

Analyses of the cytotoxicity assay data were performed using Microsoft Excel (Microsoft, Redmond, WA, USA) and GraphPad Prism version 8.0.1 for Windows (GraphPad Software, LLC, Boston, MA, USA).

The percentage of antibody construct-mediated killing was calculated as follows:

$$Killing \left( \% \right)=\left( 1-\left( \frac{mean BLI with antibody \left[ cps \right]-mean BLI Killmax \left[ cps \right]}{mean BLI control without antibody \left[ cps \right]- mean BLI Killmax \left[ cps \right]} \right) \right)*100$$

To test for possible interference of HSA, MEM α supplemented with 100 U/mL IL-2, 100 U/mL penicillin, and 100 μg/mL streptomycin was used instead of effector cell medium. This medium was prepared with either no HSA or 4.45 g/L HSA (CSL Behring GmbH, Marburg, Germany) added.

Effector cell-independent cytotoxicity mediated by Omalizumab

The direct cytotoxic activity of Omalizumab was determined using an assay adapted from the luciferase-based cytotoxicity assay described above but performed in the absence of effector cells. Ramos hεmL, Ramos hεmS and U266 were seeded at a cell density of 10^5^ cells/mL in 100 µL RPMI culture medium in white 96-well plates. Omalizumab diluted in DPBS was added in 10 µL volume to achieve final concentrations ranging from 1 mg/mL to 1 ng/mL. Each condition was tested in triplicate. Bioluminescence intensity was measured after 4, 16, and 24 hours and data were analysed as described in the previous section.

Albumin Binding of HLE-NKCE

The NK-92 cells were incubated with varying concentrations of HLE-NKCE for 20 min at 4°C. After washing, a biotinylated human albumin (Abcam) was added and the cells were incubated for another 20 min at 4°C. Following a second washing step, REAfinity Biotin-PE (Miltenyi Biotec) was added. Control samples were stained without HLE-NKCE or human albumin-streptavidin conjugate. After a final incubation and washing step, the cells were analysed via flow cytometry (LSRFortessa, BD Biosciences, Heidelberg, Germany). Data were analysed and graphs were created using FACSDiva software (BD Biosciences).

Analysis of donor variability

To characterise the BCs, flow cytometry (LSRFortessa, BD Biosciences) was employed. PBMCs from different donors were thawed and cultured in RPMI culture medium for one day. During preparation and staining, cells were washed with FACS buffer containing PBS and 0.2% bovine serum albumin (BSA; Sigma). Antibodies and reagents (Table 1) were used according to the manufacturer’s protocols. Data were analysed using FACSDiva (BD Biosciences) software.

| **Target** | **Fluorochrome** | **Clone** | **Manufacturer** | **RRID** |
| --- | --- | --- | --- | --- |
| CD3 | Pacific Blue | UCHT1 | BD Pharmingen, San Diego, CA, USA | AB_397038 |
| CD4 | VioGreen | REA623 | Miltenyi Biotec | AB_2726316 |
| CD8 | APC-Vio770 | REA734 | Miltenyi Biotec | AB_2659247 |
| CD14 | APC | REA599 | Miltenyi Biotec | AB_2655053 |
| CD16 | FITC | CLB/fcGran1 | BD Biosciences | AB_2870396 |
| CD19 | PE-Vio770 | REA675 | Miltenyi Biotec | AB_3664315 |
| CD56 | PE | REA196 | Miltenyi Biotec | AB_2726090 |
| Live/Dead | 7-AAD | -- | Miltenyi Biotec |  |

Table 1: Antibodies and viability dye used for the analysis of donor variability.

Analysis of T-cell activation

To assess the T-cell activation mediated by the TCEs, PBMCs thawed 3 days prior and maintained in RPMI culture medium were incubated at a cell density of 10^6^/mL for 2 days under different conditions: without any additive; with TransAct according to the manufacturer’s protocol as a positive control; with Ramos hεmL; with 400 ng/mL TCE; with 400 ng/mL TCE and Ramos hεmL and with 400 ng/mL TCE and Ramos hεmS. Ramos cells were added to achieve a final cell density of 10^6^ Ramos cells/mL. After 2 days of incubation, cells were washed and incubated for 20 min in 20% autologous plasma. The cells were then washed twice and stained with the premixed antibody cocktail (Table 2). Following a 20-minute incubation at approximately 22°C in the dark, the cells were washed once more, stained with 7-aminoactinomycin D (7-AAD; Miltenyi Biotec) according to the manufacturer’s protocol and analysed by flow cytometry (LSRFortessa). UltraComb eBeads Plus (Invitrogen) were used for compensation, with 7-AAD compensation performed using only 7-AAD stained cells. Data were analysed and graphs were created using FACSDiva software.

| **Target** | **Fluorochrome** | **Clone** | **Manufacturer** | **RRID** |
| --- | --- | --- | --- | --- |
| CD3 | Pacific Blue | UCHT1 | DB Pharmingen | AB_397038 |
| CD4 | VioGreen | REA623 | Miltenyi Biotec | AB_2726316 |
| CD8 | APC-Vio770 | REA734 | Miltenyi Biotec | AB_2659247 |
| CD69 | PE | FN50 | BioLegend, San Diego, CA, USA | AB_2924641 |
| CD25 | PE-Vio770 | REA570 | Miltenyi Biotec | AB_2726693 |
| CD107a | AF-647 | eBioH4A3 | eBioscience, San Diego, CA, USA | AB_657539 |

Table 2: Antibodies used in the premixed antibody cocktail for the analysis of T-cell activation.

Analysis of NK-cell activation

To assess NK-cell activation, PBMCs thawed the day before were incubated in 200 µL of RPMI culture medium at a cell density of 10^6^/mL for 12 hours under different conditions: without additive; with 25 ng/mL phorbol 12-myristate 13-acetate (PMA; Merck, Darmstadt, Germany) and 1 µg/mL ionomycin (Merck); with Ramos hεmL; with Ramos hεmL and 500 ng/mL HLE-NKCE; with 500 ng/mL HLE-NKCE. Ramos cells were added at a cell density of 5×10^5^ cells/mL. At the beginning of the incubation, an anti-CD107a-PE antibody (Miltenyi Biotec, AB_2654474) was added. Monensin (BioLegend) was added to all conditions according to the manufacturer’s protocol after 1 hour. After a 12-hour incubation at 37°C, the cells were washed and stained with a premixed antibody cocktail (Table 3). Following a 30-minute incubation at 4°C, the cells were washed, stained with 7-AAD and analysed by flow cytometry (LSRFortessa). BD CompBead Anti-Mouse Ig, κ/Negative Control Particle Set (BD Biosciences) were used for compensation, with 7-AAD compensation performed using only 7-AAD stained cells, and eGFP/FITC compensation performed with Ramos hεmL. An anti-CD38-PE (DB Pharmingen, RRID: AB_395853) was used for PE compensation, as the CD107a-PE-antibody was unsuitable for the anti-mouse Ig κ compensation beads. Data were analysed and graphs were created using FACSDiva software.

| **Target** | **Fluorochrome** | **Clone** | **Manufacturer** | **RRID** |
| --- | --- | --- | --- | --- |
| CD3 | Pacific Blue | UCHT1 | DB Pharmingen | AB_397038 |
| CD56 | PE-Cy7 | NCAM16.2 | BD Biosciences | AB_2857328 |
| CD69 | FITC | CH/4 | Caltag Laboratories, Burlingame, CA, USA | AB_10373831 |
| CD107a | PE | REA792 | Miltenyi Biotec | AB_2654474 |

Table 3: Antibodies used in the premixed antibody cocktail for the analysis of NK-cell activation.

Quantification of IgE surface expression

To estimate the number of IgE molecules expressed on the target cell surface, antibody binding capacity (ABC) was determined by quantitative flow cytometry using Quantibrite PE Beads (BD Biosciences). Ramos hεmL, Ramos hεmS and U266 cells were stained with an anti-human IgE-PE antibody (BioLegend, RRID: AB_830851) according to the manufacturer’s protocol. Following a 30 min incubation at 4°C, cells were washed and 7-AAD (Miltenyi Biotec) was added according to the manufacturer’s protocol. For compensation, the BD CompBead Anti-Mouse Ig, κ/Negative Control Particle Set (BD Biosciences) was used together with the eGFP-positive Ramos hεmL cells and 7-AAD-stained PBMCs. Samples were acquired on a BD LSRFortessa and analysed with FACSDiva software. ABC was calculated using Microsoft Excel according to the Quantibrite manual.

References

1.	Nagorsen D, Kufer P, Baeuerle PA, Bargou R. Blinatumomab: a historical perspective. *Pharmacol Ther*. 2012;136(3):334-342.

2.	DrugbankOnline. Blinatumomab. Accessed June 18, 2021. https://go.drugbank.com/drugs/DB09052

3.	Hambach J, Fumey W, Stähler T, et al. Half-Life Extended Nanobody-Based CD38-Specific Bispecific Killercell Engagers Induce Killing of Multiple Myeloma Cells. *Front Immunol*. 2022;13:838406.

4.	Behar G, Sibéril S, Groulet A, et al. Isolation and characterization of anti-FcgammaRIII (CD16) llama single-domain antibodies that activate natural killer cells. *Protein Eng Des Sel*. 2008;21(1):1-10.

5.	Dombrecht B, Schotte P, Ververken JCN, inventors. WO2012175400A1 - Serum albumin binding proteins - Google Patents. WO2012175400A1. June 14, 2012.

6.	Tom R, Bisson L, Durocher Y. Transfection of HEK293-EBNA1 Cells in Suspension with Linear PEI for Production of Recombinant Proteins. *CSH Protoc*. 2008;2008(3):pdb.prot4977.

7.	Jäger V, Büssow K, Wagner A, et al. High level transient production of recombinant antibodies and antibody fusion proteins in HEK293 cells. *BMC Biotechnol*. 2013;13:52.

8.	Winterberg KJ, Möller C, Schwarze LI, et al. Imaging BiTE-Mediated Activation of Primary Human CD8+ T Cells. *Methods Mol Biol*. 2025;2904:159-177.

9.	Harfmann M, Schröder T, Głów D, et al. CD45-Directed CAR-T Cells with CD45 Knockout Efficiently Kill Myeloid Leukemia and Lymphoma Cells In Vitro Even after Extended Culture. *Cancers*. 2024;16(2):334.

10.	Cichutek SM. *Präklinische Entwicklung adoptiver Immuntherapien für Typ-I-Allergien auf Basis chimärer Antigenrezeptoren (CARs) gegen IgE-positive B-Zellen*: Staats- und Universitätsbibliothek Hamburg Carl von Ossietzky; 2022. https://ediss.sub.uni-hamburg.de/handle/ediss/10080?mode=full

11.	Nilsson K, Bennich H, Johansson SG, Pontén J. Established immunoglobulin producing myeloma (IgE) and lymphoblastoid (IgG) cell lines from an IgE myeloma patient. *Clin Exp Immunol*. 1970;7(4):477-489.

12.	Brightbill HD, Jeet S, Lin Z, et al. Antibodies specific for a segment of human membrane IgE deplete IgE-producing B cells in humanized mice. *J Clin Invest*. 2010;120(6):2218-2229.

13.	Ikeyama S, Nakagawa S, Arakawa M, Sugino H, Kakinuma A. Purification and characterization of IgE produced by human myeloma cell line, U266. *Mol Immunol*. 1986;23(2):159-167.
